# The effect of amino acid deletions and substitutions in the longest loop of GFP

**DOI:** 10.1186/1472-6769-7-1

**Published:** 2007-06-26

**Authors:** Gabriela Flores-Ramírez, Manuel Rivera, Alfredo Morales-Pablos, Joel Osuna, Xavier Soberón, Paul Gaytán

**Affiliations:** 1 Departamento de Ingeniería Celular y Biocatálisis. Instituto de Biotecnología, Universidad Nacional Autónoma de México, Ap. Postal 510-3 Cuernavaca, Morelos 62250, México

## Abstract

**Background:**

The effect of single and multiple amino acid substitutions in the green fluorescent protein (GFP) from *Aequorea victoria *has been extensively explored, yielding several proteins of diverse spectral properties. However, the role of amino acid deletions in this protein -as with most proteins- is still unknown, due to the technical difficulties involved in generating combinatorial in-phase amino acid deletions on a target region.

**Results:**

In this study, the region I129-L142 of superglo GFP (sgGFP), corresponding to the longest loop of the protein and located far away from the central chromophore, was subjected to a random amino acid deletion approach, employing an in-house recently developed mutagenesis method termed Codon-Based Random Deletion (COBARDE). Only two mutants out of 16384 possible variant proteins retained fluorescence: sgGFP-Δ I129 and sgGFP-Δ D130. Interestingly, both mutants were thermosensitive and at 30°C sgGFP-Δ D130 was more fluorescent than the parent protein. In contrast with deletions, substitutions of single amino acids from residues F131 to L142 were well tolerated. The substitution analysis revealed a particular importance of residues F131, G135, I137, L138, H140 and L142 for the stability of the protein.

**Conclusion:**

The behavior of GFP variants with both amino acid deletions and substitutions demonstrate that this loop is playing an important structural role in GFP folding. Some of the amino acids which tolerated any substitution but no deletion are simply acting as "spacers" to localize important residues in the protein structure.

## Background

The green fluorescent protein (GFP) has revolutionized molecular and cell biology, because it can be used as a reporter of gene expression and protein localization due to its inherent capacity to generate an efficiently emitting internal fluorophore [1-3]. GFP is a 28 kDa protein composed of 238 amino acid residues. X-ray crystal studies of GFP uncovered a β-barrel structure resembling a soda can. The wall of the β-can structure is built by 11 antiparallel β-strands. This β-sheet secondary structure surrounds a single central α-helix that contains the fluorophore, spontaneously formed by post-translational modification of residues Ser65, Tyr66 and Gly67. Two protein lids, composed mainly of residues 74–91 and 128–145, cover the β-can structure and isolate the chromophore from the surrounding solvent. Because of the simplicity of the chromophore formation, modifications on the primary structure of GFP have produced several improved variants, either more fluorescent [4] or blue/red-shifted [5]. These changes have been achieved employing site-directed approaches [6], regional combinatorial approaches [4] and fully random approaches such as DNA shuffling [7].

High resistance to proteolysis [3], detergents [8], heat [9] and denaturing agents [9] are consequences of the rigid structure of GFP which seems to be a nearly size-minimized protein [10]. GFP tolerates enlargement through the insertion of short peptides [11], long peptides [12] and even complete proteins [13] in different locations, but it is particularly sensitive to shortening by internal site-directed deletions [10,11]. Prior to this study, only amino- or carboxyl-terminus deletions have been reported for GFP [10].

The role of insertions and deletions (indels) in protein evolution is likely to be very significant, as can be inferred by inspecting any sequence or structure alignment of homologous proteins. Unfortunately, their role has been difficult to assess experimentally, due to lack of convenient methods to generate indels systematically. Recently, we described a novel and unique mutagenesis method (named COBARDE) capable to generate codon-based random amino acid deletions on interesting protein regions [14]. Using COBARDE, the specificity of the enzyme TEM-1 β-lactamase was modified by random combination of several amino acid deletions located around the active site.

**Figure 1:**
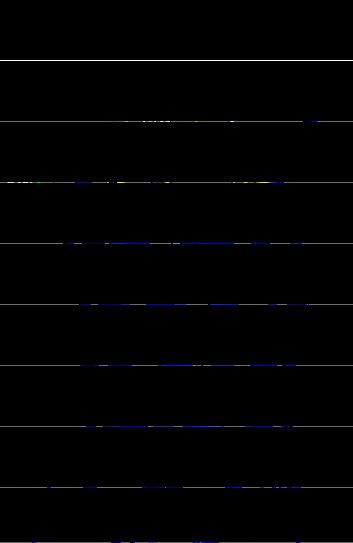
**Structure of wild-type GFP (1EMA)**. The region 128–141 (129–142 in sgGFP) explored in this study is shown in yellow. N-terminal and C-terminal are marked with capital letters and the chromophore located in the center of the barrel is also shown in yellow.

To extend the evaluation of COBARDE as a potential tool in protein engineering, particularly to explore the relationship between protein size and function, a systematic search of deletions in the region 129–142 of superglo Green Fluorescent Protein (sgGFP) was undertaken in the present study. This region is equivalent to amino acids 128–141 of wild-type GFP from *Aequorea victoria *and corresponds to the longest loop of the protein (see Fig. 1).

## Results and discussion

COBARDE was originally tested on TEM-1 β-lactamase with interesting results [14]. There were clear indications that this enzyme was able to tolerate even long internal deletions [15] and this was confirmed by the systematic introduction of deletions. GFP is, however, completely different because no active internal deletions have been yet reported. We thought an excellent test bed for COBARDE was to attempt to shorten this already rather rigid and structurally compromised protein.

We selected the region located between residues 129–142 as target of the mutagenesis for three reasons: 1) It is the longest loop of the protein; 2) two previous attempts of deletions in this area failed to produce fluorescent proteins [10,11]; 3) Published sequence alignments of GFP versus GFP-like proteins of anthozoas suggest that GFP may tolerate deletion of either G138 [16] or H139 [17] (G139 and H140 respectively in sgGFP).

**Figure 2:**
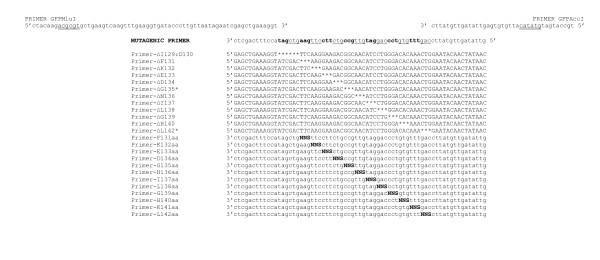
**Oligonucleotides used for preparation of sgGFP mutants as described in M&M**. For clear visualization, codons subjected to combinatorial deletion are alternatively labeled in bold and underlined. Asterisks represent nucleotide deletions. N represents an equimolar mixture of the four nucleotides and S a mixture of G and C. Restriction sites are double-underlined.

Experimental work started with synthesis of the oligonuclotide library. One current limitation of Fmoc-based mutagenesis methods is depurination of benzoylated deoxyadenosines (dA^bz^s), giving rise to a high ratio of backbone cleavage (our own unpublished results). This depurination problem is magnified if the target sequence is dA-enriched at the 3' end, because synthesis proceeds from 3' towards 5' direction. The severity of the problem prevented a successful synthesis of the coding strand for the targeted region. Thus, we resorted to synthesizing a complementary sequence, further modified to reduce even more the content of dA^bz^s near the 3'end (indicated in bold face): 3' ctc **g**a**c **ttt cca TA**G **CTG AAG TTC CTT CT**G **CCG TTG TA**G **GAC CCT GTG TTT **G**AC ctt atg ttg ata ttg 5'. This sequence contains 17 fewer dA^bz^s than the original sequence and was successfully assembled by COBARDE. The oligonucleotide was used as a PCR template of two partially complementary primers to generate a 148 bp double stranded fragment that included the *Mlu *I and *Acc *I restriction sites as shown in Figure 2. The product was digested, and ligated to the kanamycin-carrying cloning vector pT4GFP^Mlu ^(see M&M for preparation of this recipient plasmid). The ligation mixture was transformed into XL1-Blue cells to give a library of 2 × 10^6 ^variants. Analysis of colonies grown on plates for 24 h at 37°C revealed that more than 99% of the transformants were non-fluorescent to the naked eye, indicating that most of the deletions perturbed protein structure and/or function. Plasmid DNA from 40 randomly chosen fluorescent clones was obtained and sequenced revealing that 14 of the samples corresponded to re-ligated vector due to incomplete *Mlu *I/*Acc *I digestion; 22 corresponded to wild-type sgGFP created with the wild-type oligonucleotide generated in the library and only 4 of the clones were mutants that retained fluorescence. These mutants corresponded to single amino acid deletions of isoleucine 129 (sgGFP-Δ I129) and aspartate 130 (sgGFP-Δ D130). Each mutant was found twice.

On the other hand, the DNA sequence analysis of 33 non-fluorescent colonies (Table 1) gave an estimation of the quality of the library and provided insights into the kind of mutations that destroy fluorescence. From the data shown in Table 1 we draw the following conclusions:

**Table 1:** DNA sequence of non-fluorescent clones chosen randomly from the library generated with COBARDE

Clone	*sgGFP *modified region														Codons deleted
		
	Ile 129	Asp 130	Phe 131	Lys 132	Glu 133	Asp 134	Gly 135	Asn 136	Ile 137	Leu 138	Gly 139	His 140	Lys 141	Leu 142	
Wt															0
1	ATC		TTC				GGC	AAC							10
2	ATC	GAC	TTC	AAG	GAA	GAC	GGC	AAC	ATC	CTG	GGA	CAC		CTG	1
3	ATC	GAC	TTC					AAC			GGA	CAC	AAA	CTG	6
4	ATC		TTC	AAG				AAC	ATC	CTG	GGA	CAC		CTG	5
5	ATC	GAC	TTC	AAG	GAA	GAC	GGC	AAC		CTG					5
6	ATC							AAC	ATC		GGA	CAC		CTG	8
7	ATC		TTC			GAG	GGC							CTG	9
8	ATC	GAC				GAC		AAC		CTG					9
9	ATC	GAC		AAG	GAA	GAC	GGC	AAC			GGA		AAA	CTG	4
10	ATC	GAC	TTC	AAG				AAC		CTG	GGA			CTG	6
11	ATC	GAC			GAA	GAC			ATC						9
12	ATC	GAC				GAC	GGC		ATC	CTG	GGA				7
13	ATC	GAC	TTC	AAG	GAA	GAC	GGC	AAC				CAC		CTG	4
14	ATC	GAC		AAG	GAA		GGC	AAC	ATC	CTG		CAC	AAA	CTG	3
15	ATC	GAC		AAG	GAA	GAC				CTG					8
16	ATC	GAC			GAA	GAC	GGC		ATC			CAC		CTG	6
17	ATC	GAC		AAG	GAA	GAC		AAC							8
18	ATC	GAC						AAC					AAA	CTG	9
19	ATC	GAC	TTC			GAC	GGC		ATC	CTG	GGA		AAA	CTG	4
20	ATC	GAC	TTC	AAG		GAC								CTG	8
21	ATC	GAC	TTC		GAA	GAC	GGC	AAC			GGA	CAC	AAA	CTG	3
22	ATC	GAC	TTC					AAC		CTG			AAA	CTG	7
23	ATC			AAG		GAC				CTG					10
24	ATC		TTC	AAG			GGC	AAC							9
25	ATC		TTC		GAA		GGC		ATC	CTG				CTG	7
26	ATC	GAC	TTC		GAA	GAC	GGC	AAC			GGA	CAC	AAA	CTG	3
27	ATC	GAC	TTC		GAA		GGC			CTG					8
28	ATC	G	TTC		GAA		GGC	AAC		CTG		CAC		CTG	6
29	ATC	GAC		AAG			GGC	AAC	ATC		GGA			CT	6
30	ATC		T	AAG		GAC		AAC		CTG	GGA		AAA	CTG	5
31	AC	GAC							ATC	CTG	GGA	CAC	AAA	CTG	6
32	ATC					GA	GGC	AAC		CTG	GGA		AAA		7
33	ATC	GAC		AAG				AAC		CTG	GA				8
oligo	TAG	CTG	AAG	TTC	CTT	CTG	CCG	TTG	TAG	GAC	CCT	GTG	TTT	GAC	

Empty spaces represent deleted codons. Last row contains the oligonucleotide segment that was subjected to mutagenesis. Dots represent single nucleotide deletions. Nucleotide insertions are shown in gray letters.

1) A successful mutagenesis (with an average 50% mutagenesis rate) was achieved on the target region. It is clear from Table 1 that amino acid deletions were well spread and represented along the target, except for the first codon (encoding I129) which was mutated at only 2% rate because the Fmoc-Cl delivering line was not properly primed. However, this failure was corrected from the second codon on.

2) 6 out of 33 clones (clones 28–33) contained either single nucleotide deletions or insertions that change the open reading frame of the genes. Although this ratio of undesired variants is apparently high (18%), it is within the error range found in conventional oligonucleotides as has been observed during assembly of synthetic genes [18-20]. Single nucleotide deletions usually occur because of incomplete capping step during each synthesis cycle. This chemical imperfection may be significantly reduced with the use of UNICAP [21], a recently commercially available potent capping reagent. However, the remaining 1.68 × 10^6 ^useful variants (82%) are enough to represent the complete set of 16384 (2^14^) possible deletion variants. Considering an average 0.5 mutagenesis rate per codon, each of the mutants should be represented with the same frequency and we only need a library of 75492 clones to find the least represented variant with 99% confidence [22]. Further, since the wild-type clone was found several times in the fluorescence screening, it can be concluded that all mutants were well represented in the experimental library.

3) The library follows a roughly binomial distribution. Mutants carrying 6, 7, 8 and 9 deletions were the most frequent.

4) Most of the deletions in the explored loop destroy GFP fluorescence. This result agrees with those found by Li *et al *[10] and Kitamura *et al *[11]. Li removed the region comprising amino acids 132–139 of GFP by site-directed mutagenesis, whereas Kitamura randomly removed tri-peptide blocks in the region 125–142. Both studies found the deletions to cause a complete loss of fluorescence.

Our sample of 33 non-fluorescent mutants sequenced included only one single deletion mutant, sgGFP-Δ K141, yet two single deletions, sgGFP-Δ I129 and sgGFP-Δ D130, conserved fluorescence. To make sure that our fluorescence screening was able to pick up all active robust mutants, we decided to individually create the remaining 11 single deletion mutants and the double mutant that combines Δ I129 and Δ D130 by site-directed mutagenesis using the specific oligonucleotides shown on Figure 2.

Confirming the validity of the library screening, none of the *E. coli *expressing these mutants displayed a green-fluorescent phenotype on plates, after incubation at 37°C for 24 h. Fluorescence scanning of cultures containing each of the fourteen single deletion mutants and the double mutant, grown for 12 h at 37°C, confirmed the results observed in plates. These experiments also discarded the hypothesis that sgGFP**-**ΔG139 and sgGFP**-**ΔH140 may be functional, as suggested by the alignments of GFP versus GFP-like proteins.

Other aligments based in three-dimensional structures of GFP versus GFP-like proteins suggest that region 128–141 does not tolerate deletions and that GFP must tolerate deletion of Y143 [23,24]. To test the confidence of these 3D aligments for protein engineering, we removed the equivalent residue (Y144) in sgGFP by site-directed mutagenesis and the fluorescence was completely lost. The conclusion of these aligments is obvious, no prediction can be done when the sequence identity between the proteins compaired is so low. The sequence identity between GFPs and GFP-like proteins is around 25%.

**Figure 3:**
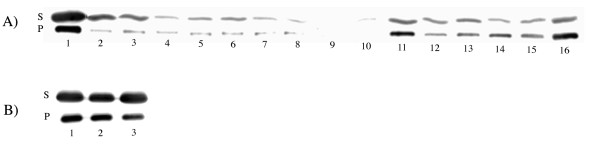
**Western blotting analysis of deleted mutants grown at two different temperatures**. (A) Single amino acid deletions comprised in the region 129–142 of sgGFP and the double mutant sgGFP-ΔI129/ΔD130 grown at 37°C. (B) Some sgGFP mutants grown at 30°C. S and P represent the soluble and insoluble fraction of the cells, respectively. Fractions S and P were run on different gels. Lane 1: sgGFP wt, lane 2: sgGFP-ΔI129, lane 3: sgGFP-ΔD130, lane 4: sgGFP-ΔF131, lane 5: sgGFP-ΔK132, lane 6: sgGFP-ΔE133, lane 7: sgGFP-ΔD134, lane 8: sgGFP-ΔG135, lane 9: sgGFP-ΔN136, lane 10: sgGFP-ΔI137, lane 11: sgGFP-ΔL138, lane 12: sgGFP-ΔG139, lane 13: sgGFP-ΔH140, lane 14: sgGFP-ΔK141, lane 15: sgGFP-ΔL142 and lane 16: sgGFP-ΔI129/ΔD130.

Additional characterization of whole cells containing the mutants sgGFP-Δ I129 and sgGFP-Δ D130 revealed that both proteins suffered a blue-shift of two nanometers in their maximum emission and their fluorescence intensity was reduced to 21% and 17%, respectively, relative to wt sgGFP. The last result did not correlate with the phenotype observed in plates, where the green color of the mutants was only slightly less intense than the wild-type protein. We then decided to measure the quantum yield of the mutant proteins, which turned out to be 31% and 21% smaller than the parent protein, respectively. Because the quantum yield decrement of the mutants did not fully account for the fluorescence loss, we turned our attention towards protein concentration in the cells, another factor that affects fluorescence intensity. The amount of soluble and non-soluble protein for each mutant was analyzed by western blotting as shown in Figure 3, using anti-GFP for the detection. This experiment clearly revealed that the main reason for the reduction or loss of fluorescence of the mutants was their low concentration which, in turn, could also be due to low stability or incorrect folding [25]. Not surprisingly, sgGFP-Δ I129 and sgGFP-Δ D130 were the best mutants expressed. To assess if the proteins were inactivated by improper folding we grew the mutants at 30°C. At this lower temperature, the fluorescence of sgGFP-Δ I129 increased from 21% to 46%, whereas sgGFP-Δ D130 increased from 17% to 116% as compared to wt sgGFP. These results indicated that both deletion mutants are thermosensitive, and even more, at lower temperatures sgGFP-Δ D130 is more fluorescent than the wild-type protein. Lower temperatures frequently favor appropriate folding of mutants [13]. Western blotting of the mutants grown at 30°C, shown on Figure 3b, confirmed that the protein concentration was increased.

It is worth mentioning that plated colonies expressing the other single deletion mutants remained being non-fluorescent neither at 30°C nor at 22°C during 15 days of growing.

**Figure 4:**
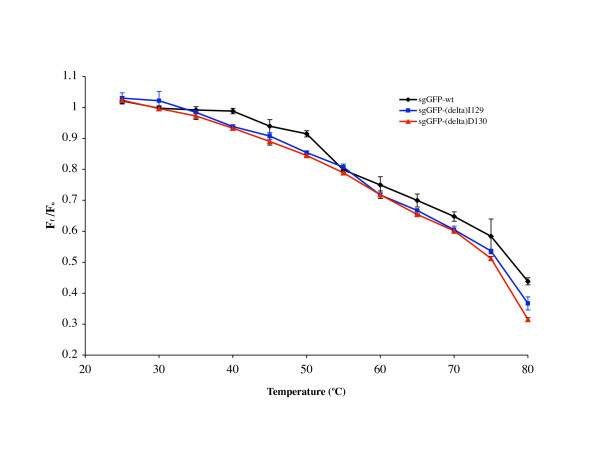
**"Temperature denaturation curves of the purified proteins sgGFP wt, sgGFP-ΔI129 and sgGFP-ΔD130**. Triplicated aliquots of each sample were subjected to different temperatures (25°C, 30°C, 35°C, 40°C, 45°C, 50°C, 55°C, 60°C, 65°C, 70°C, 75°C and 80°C) during 5 minutes and the initial (F_o_) and final fluorescence (F_f_) was measured. The ratio F_f_/F_o _versus temperature was plotted."

Temperature denaturation curves (see Figure 4) for the active purified mutants sgGFP-Δ I129 and sgGFP-Δ D130, demonstrated that these proteins are less heat stable than the parent protein, but not enough to give account for the significative protein reduction at 37°C. Therefore, these amino acids are essential for good folding, especially D130.

In the case of some non-functional mutants such as sgGFP-Δ L138 and sgGFP**-**ΔI129/ΔD130, low protein concentration was not the only explanation for their loss of fluorescence. These two mutants gave rise to significant inclusion bodies but still a considerable amount of protein remained in solution, which would be expected to give a signal if the proteins were fluorescent *per se*. We believe these mutants are correctly folded but maturation of the chromophore is impaired, in a mode similar to the colorless GFP isolated from *Aequorea corulescens *(acGFP) or the enhanced mutant aceGFP-G222E [26]. More biophysical and biochemical assays are needed to elucidate which process(es) are affected – cyclization, oxidation or dehydration.

The most important conclusion resulting from the deletion studies reported hereby is the key role of residues 131–142 (130–141 in GFP) for appropriate folding of the protein. This result agrees with results reported by Baird *et al *[13] working with permutations. They found that GFP can be opened in different locations only after residue N144 but they did not explain the absence of openings in the first half of the protein. Therefore, the region 131–142 seems to be acting as a bridge that joins two parts of the protein independently folded.

To further explore how essential is the sequence at the sub-region 131–142 we decided to subject each of the twelve positions to single site-saturation mutagenesis (see M&M for details) using the degenerated oligonucleotides shown on Figure 2. To our surprise, most of the variants (55%) found in the libraries of substitutions displayed a green-fluorescent phenotype in plates, after 24 h of growth at 37°C, showing that substitutions are tolerated where deletions are not. DNA sequence data from several randomly chosen fluorescent and non-fluorescent colonies (as appeared in the plate assay) are concentrated in Table 2. The data show that G135 is the least tolerant amino acid, with allowed replacements of this residue only producing pale green-fluorescent colonies (due to diminished soluble protein in the cells; data not shown). This buried amino acid forms part of a short α-helix located at the center of the loop. Apparently, the major function of this α-helix is to position I137 towards the heart of the barrel in order to fix part of the loop.

**Table 2:** Analysis of fluorescent and non-fluorescent mutants carrying single amino acid substitutions

WT aa	AS	Hydrophobic aa									Hydrophilic aa						Acid		Basic			Stop
		
		W	F	P	M	L	I	V	A	G	N	Q	S	T	C	Y	D	E	R	K	H	X
F131	B			*1*		**1**	**1**		2*			*1*		*1*			*1*			*1*	*1*	
K132	E	**1**					**1**		**1**	**1**								**1**	**2**			
E133	E			**1**		**2**		**1**	**3**	**1**	**1**		**1**	**1**			**1**		**3**			
D134	E		**1**									**1**	**2**	**2**					**2**			
G135	B					1	1*	1*		**1**			1*		1	1						
N136	E		**1**	**4**	**1**	**1**		**4**	**4**			**1**		**2**					**1**	**1**	**1**	
I137	B					**4**		**2**		*1*			1*				*1*	*2*	*2*			*1*
L138	B		**1**		**2**	**2**	**1**			1*					1				1			
G139	E			1*		**2**			**1**	**1**	**1**	**2**	**2**	**1**	**1**					**2**	**1**	
H140	E			*1*			1*				**1**					**1**			**2**	**2**		
K141	E			2*	1				**1**		**1**			**1**					**1**		**1**	
L142	B	**1**					**1**	**1**		*2*	*1*		*1*				*1*					

Mutants displaying a green-fluorescent phenotype to the naked eye are shown in bold numbers. Mutants displaying a white phenotype are shown in italic numbers. Mutants displaying a pale green-fluorescent phenotype are shown in underlined numbers. Those mutants labeled with asterisk turned pale green-fluorescent after prolonged growing at room temperature. The number represents the number of times that each mutant was found. In this table, empty spaces represent wild-type amino acids. B means buried amino acid; E, solvent exposed amino acid. During screening of fluorescent mutants, seven clones corresponded to wt sgGFP generated with the mixtures of degenerated oligonucleotides.

Positions 131, 137, 138 and 142 only tolerated conservative replacements with hydrophobic residues. Because F131, L138 and L142 are also buried in the core of the protein, these amino acids are likely to be important for fixation of the loop. The non-fluorescent mutant F130A also revealed that size of the hydrophobic side-chain is important for appropriate packaging of the protein. On the other hand, residues H140 and K141 were replaced only with hydrophilic amino acids, suggesting ionic or H-bond interactions with neighbor amino acids. Finally, residues K132, E133, D134, N136 and G139, whose side-chains are exposed to the solvent, tolerated any amino acid substitution.

Our results with substitutions confirmed the scope of the scanning mutagenesis approach to identify buried and exposed amino acids in proteins of unknown structure as proposed by Bajaj *et al *[27]. For instance, substitution of buried amino acids with charged residues, as in mutants F131D, F131K, I137D, I137E, I137R and L142D, destroyed fluorescence, apparently because of protein instability. However, it is important to note that some mutants (labeled with asterisk in Table 2), initially non-fluorescent in the plate assay, turned pale green-fluorescent after 3–5 additional days of growing at room temperature, suggesting slow maturation of the chromophore as in the case of acGFP [26].

## Conclusion

COBARDE has been demonstrated to be a powerful and confident mutagenesis tool to reduce, although to a minimal amount, a particularly constrained structure and rigid protein such as GFP. The method allowed us to select two unique active mutants out of 16384 possible variant proteins in a stretch of 14 amino acids. If this achievement is extrapolated to enzymes, then the optimal mutant could be easily obtained using appropriate selection conditions. The radical difference between the complete exploration of random deletions versus random substitutions is the library size. For instance, if the same stretch of 14 amino acids is subjected to substitution, using a mixture of 20 trimers to saturate each of the wild-type codons, a library of 1.6 × 10^18 ^(20^14^) variants would be generated. The complete transformation of this amount is impossible to achieve and, consequently, one would never be sure if the best mutant has been expressed and selected in the biological libraries. Even more, COBARDE might be easily extended to explore regions up to 18 amino acids with a practical transformation efficiency of 10^7 ^variants.

On the other hand, the systematic generation of single amino acid deletions and single substitutions on the region 129–142 of sgGFP demonstrated that some residues, highly tolerant to substitutions but intolerant to deletions, play a simple "spacer" role to locate near residues in appropriate positions of the protein. It also confirmed that deletions are more disruptive events than substitutions, affecting mainly the protein folding and stability. Knowing these apparently adverse results, the question is obvious: is it really worth studying deletions? The answer is clearly YES. COBARDE might be used to reduce the size of several therapeutic proteins in order to modify their diffusion in the body and might also be used to shorten or enlarge the active site of enzymes (depending on the region subjected to mutagenesis) in order to accept smaller or larger substrates than the natural ones.

## Methods

### Oligonucleotide synthesis

The Codon-Based Random Deletion (COBARDE) method relies on oligonucleotide synthesis. Briefly, COBARDE consists of arresting part of the growing oligo in the preceding nucleotide to the target codon to be deleted. The partial arresting is performed by substoichiometric reaction of the growing oligo with a diluted solution of fluorenylmetoxycarbonyl-chloride (Fmoc-Cl), in the presence of 4,4-dimethylaminopiridine (DMAP) as catalyst. The Fmoc protecting group is known to be stable to acid and labile to alkali. In the following step, the wild-type codon is assembled by performing three consecutive couplings of dimethoxytrityl (DMTr) protected monomers with the unblocked oligo. Both, Fmoc and DMTr groups are subsequently removed using alkali and acid treatment respectively, and the growing oligo is ready for another deletion cycle. If the substoichiometric reaction is high, the library will be enriched with mutants carrying several codon deletions, but if the substoichiometric reaction is low the library will be enriched with mutants carrying few codon deletions [14].

For this work, the antisense oligonucleotide library 5' gttatagttgtattc CA**G **TTT GTG TCC CAG **G**AT GTT GCC **G**TC TTC CTT GAA GTC **G**AT accttt**c**a**g**ctc 3' encoding amino acids I129-L142 of sgGFP was synthesized by COBARDE. This sequence corresponds to nucleotides 373–441 of *sgGFP *minus strand (equivalent to nucleotides 370–438 in *GFP*). The codons subjected to random deletion are shown in capital letters and the non-modified flanking regions in lower case. This sequence also included six silent nucleotide substitutions (bold letters) that change t376c, a378g, t387c, t402c, t411c and t424c in the coding strand. Such modifications were done to differentiate wild-type clones generated in the mutant library from wild-type clones obtained by re-ligation of the recipient plasmid and to reduce the dA ratio in the oligo. The Fmoc-Cl solution used to repeatedly block part of the growing oligo was 12 mM which produces approximately 50% deletion mutants per site.

All other ancillary oligonucleotides used in this research were synthesized at the core facility of our Institute, as recommended by the DNA synthesizer manufacturer (Applied Biosystems, Inc.).

### Construction of the recipient plasmid pT4GFPMlu

sgGFP is an engineered variant (F64L, S65C, I167T) of GFP, being brighter than wild-type GFP because of its greater solubility and folding at 37°C, displaying unique excitation and emission peaks at 474 nm and 509 nm respectively [28]. *sgGFP *gene from vector pQBI25 (Q-BIOgene) was subjected to two site-directed mutagenesis steps to perform the silent nucleotide substitutions t234c and a327g, following the procedure published by Merino *et al *[29]. The first change destroys an *Nde *I restriction site found in the gene and the latter creates an *Mlu *I restriction site, with no alteration of amino acid sequence. The modified gene was amplified with two external primers containing *Nde *I and *Xho *I restriction sites and was cloned into a pT4 cloning vector [30] under control of the trc promoter. This construction was finally digested with the restriction enzymes *Mlu *I and *Acc *I (New England Biolabs) to prepare the recipient cloning vector pT4GFP^Mlu^.

### Mutagenesis, cloning and selection of GFPs carrying random amino acid deletions

Mutant cassettes were generated by extension of the primers GFP-MluI (5'ctacaagacgcgtgctgaagtcaagtttgaaggtgatacccttgttaatagaatcgagctgaaaggt 3') and GFP-AccI (5'tgccatgatgtatacattgtgtgagttatagttgtattc3'), using the oligonucleotide library as template (Figure 2); these primers contain the *Mlu *I and *Acc *I restriction sites, respectively, for cloning purposes. 50 pmol of each primer and 5 pmol of the oligonucleotide library were subjected to PCR. TaqGold polymerase (Applied Biosystems) was used for the PCR, following the conditions: 1×: 95°C for 5 min and 15X: 94°C for 30 sec, 30°C for 30 sec, 72°C for 30 sec.

The extended fragment was purified by agarose gel and digested with *Mlu *I and *Acc *I restriction enzymes. The digested product was ligated overnight to 3 pmol of the recipient plasmid pT4GFP^Mlu ^using T4 DNA ligase (New Englad Biolabs). The ligation mixture was electroporated into XL1-Blue cells. A 1/1000 aliquot of the transformants was plated on a kanamycin-containing LB plate, incubating for 24 h at 37°C to quantify the library size. The remaining transformants were grown overnight into 20 ml of kanamycin-containing LB to recover the transformants as a library of plasmids.

The pool of plasmids was re-electroporated into XL1-Blue cells and the transformants were grown on kanamycin-containing LB plates at 37°C for 24 h. 40 colonies displaying a green-fluorescent phenotype to the naked eye were randomly chosen and sequenced. 33 white colonies were also randomly chosen and sequenced to analyze the diversity of mutations generated in the library, as well as the type of deletions that destroy fluorescence.

### Amino acid deletions created by site-directed mutagenesis

The site-directed mutants sgGFP**-**ΔF131, sgGFP**-**ΔK132, sgGFP**-**ΔE133, sgGFP**-**ΔD134, sgGFP**-**ΔG135, sgGFP**-**ΔN136, sgGFP**-**ΔI137, sgGFP**-**ΔL138, sgGFP**-**ΔG139, sgGFP**-**ΔH140 and sgGFP**-**ΔL142, which delete one amino acid each, as indicated in their names, were constructed following the standard protocol described for the library of GFPs carrying random amino acid deletions, replacing the oligonucleotide library by each of the specific primers shown on Figure 2. The double mutant sgGFP**-**ΔI129:ΔD130 that combines deletion of I129 and D130 was also assembled. Each mutant was confirmed by DNA sequencing.

### Random single amino acid substitutions generated by site-directed mutagenesis

Single substitution of residues F131, K132, E133, D134, G135, N136, I137, L138, G139, H140, K140 and L142 with any of the other amino acids (aa) was performed by the twelve independently synthesized degenerated oligonucleotides shown on Figure 2. These primers were grouped into three sets of four oligonucleotides and cloned as described above for the mutagenic oligonucleotide. Several colonies from each set, displaying a green fluorescent phenotype, pale-green phenotype or white phenotype to the naked eye after 24 hours of growing at 37°C, were randomly chosen and analyzed by DNA sequencing.

### Immunoblotting and fluorescence analysis of sgGFPs

XL1-Blue cells expressing the appropriate sgGFP protein (either wild-type or mutant) were inoculated into kanamycin-supplemented liquid LB and the cultures were grown overnight, under shaking at 37°C. Whole cell extracts were prepared from the cultures normalized at the same optical density (OD_600*nm*_). The pellet was obtained by centrifugation and resuspended in a B-PER (nonionic detergent in 20 mM Tris-HCl, pH 7.5) bacterial protein extraction reagent (PIERCE). A protease inhibitor cocktail, complete EDTA-free (Roche), was added as recommended. Insoluble and soluble protein fractions were obtained by centrifugation. Total protein concentration was measured by Bradford reagent (BIORAD). For Western blotting analysis, 20 μg of proteins of the soluble and insoluble fractions were resolved by 12% SDS-PAGE and then transferred to nitrocellulose membranes (Amersham Pharmacia Bioscience) for 1 hour at 70 mA in a semi-dry transfer unit (Hoefer SemiPhor-Amersham Pharmacia Biotech). Western blotting was performed following standard protocols [31], using anti-GFP (Clontech) for the detection.

The fluorescence assay was recorded on a Perkin Elmer Luminescence Spectrometer LS50B or a Genesis workstation TECAN using the module *safire*. All measurements were made in triplicate.

For determination of the quantum yield of mutants sgGFP-ΔI129 and sgGFP-ΔD130, a calibration curve of absorbance at 474 nm versus fluorescence emission at 508 nm was performed for lysates of wild-type sgGFP, using the luminescence spectrometer LS50B (Perkin Elmer). Quantum yield of the active mutants carrying deletions, relative to wild-type sgGFP, was calculated by determining the absorbance and fluorescence of cell extracts of one sample and extrapolating to the calibration curve. All measurements were made in triplicate.

## Authors' contributions

GFR, MR, JO and AMP carried out the mutagenesis studies. XS participated in the design of the study and drafted part of the manuscript. PG conceived of the study, and participated in its design and coordination and drafted most of the manuscript. All authors read and approved the final manuscript.
